# Repurposing iron chelators for accurate positron emission tomography imaging tracking of radiometal‐labeled cell transplants

**DOI:** 10.1002/mco2.473

**Published:** 2024-01-30

**Authors:** Qian Xu, Xinyu Wang, Ziqian Mu, Yixiang Zhou, Xiang Ding, Xin Ji, Junjie Yan, Donghui Pan, Chongyang Chen, Yuping Xu, Lizhen Wang, Jing Wang, Guangji Wang, Min Yang

**Affiliations:** ^1^ Department of Radiopharmaceuticals School of Pharmacy Nanjing Medical University Nanjing China; ^2^ NHC Key Laboratory of Nuclear Medicine Jiangsu Key Laboratory of Molecular Nuclear Medicine Jiangsu Institute of Nuclear Medicine Wuxi China; ^3^ Jiangsu Renocell Biotech Co., Ltd. Nanjing China; ^4^ Key Laboratory of Drug Metabolism and Pharmacokinetics State Key Laboratory of Natural Medicines China Pharmaceutical University Nanjing China

**Keywords:** cell therapy, cell tracking, iron chelator, PET imaging, radiolabel

## Abstract

The use of radiolabeled cells for positron emission tomography (PET) imaging tracking has been a promising approach for monitoring cell‐based therapies. However, the presence of free radionuclides released from dead cells during tracking can interfere with the signal from living cells, leading to inaccurate results. In this study, the effectiveness of the iron chelators deferoxamine (DFO) and deferiprone in removing free radionuclides ^89^Zr and ^68^Ga, respectively, was demonstrated in vivo utilizing PET imaging. The use of DFO during PET imaging tracking of ^89^Zr‐labeled mesenchymal stem cells (MSCs) significantly reduced uptake in bone while preserving uptake in major organs, resulting in more accurate and reliable tracking. Furthermore, the clearance of free ^89^Zr in vivo resulted in a significant reduction in radiation dose from ^89^Zr‐labeled MSCs. Additionally, the avoidance of free radionuclide accumulation in bone allowed for more precise observation of the homing process and persistence during bone marrow transplantation. The efficacy and safety of this solution suggest this finding has potential for widespread use in imaging tracking studies involving various cells. Moreover, since this method employed iron chelator drugs in clinical use, which makes it is a good prospect for clinical translation.

## Graphical abstract

PET imaging tracks radiolabeled cell therapies, but dead cells release free radionuclides, confounding live cell signals. Iron chelators deferoxamine and deferiprone cleared radionuclides ^89^Zr and ^68^Ga in vivo, enabling accurate radionuclides‐mesenchymal stem cell tracking and reduced radiation. Chelation avoided radionuclide bone accumulation, enabling precise cell homing observation. This clinically translatable method promises widespread utility for cell tracking studies.

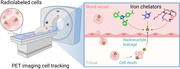

## 1 INTRODUCTION

Cell therapies have captured tremendous attention in the treatment of a range of diseases, including cancer, infections, autoimmune diseases, and neurodegenerative diseases.^1^, ^2^, ^3^ These therapies encompass various modalities, and remarkable progress has been achieved so far, including chimeric antigen receptor T cells (CAR‐T), T‐cell receptor T cells (TCR‐T), tumor‐infiltrating lymphocytes (TILs), natural killer cells (NK), stem cells transplant, and bone marrow cells transplant.^4^, ^5^, ^6^, ^7^ Being a class of “living” drugs, some of the pharmacokinetic (PK) parameters of cell therapies are challenging to assess through conventional drug analysis. The study of absorption, distribution, metabolism, and excretion (ADME) and PK properties are crucial in drug discovery and development.^8^, ^9^ However, there are obstacles in the ADME and PK evaluation of adoptively transferred cells. For genetically modified cells, PK studies are performed using flow cytometry and quantitative polymerase chain reaction (qPCR) techniques,^10^, ^11^, ^12^ while for other cells, such methods may not be available. The human‐specific hAlu gene can be used to determine the PK of Mesenchymal stem/stromal cells (MSCs) by qPCR analysis, but only murine xenograft models.^13^, ^14^ This approach cannot be directly translated to the clinic due to interference from homologous genomic elements in human patients. Alternative strategies would need to be explored to evaluate PK of adoptively transferred MSCs in the autologous setting. Moreover, these invasive techniques typically necessitate ex vivo tissue samples, rendering it challenging to measure samples other than blood for clinical applications. As a result, there is a pressing clinical need for innovative noninvasive techniques for PK evaluation.

Radiolabeling and nuclear imaging tracking of adoptively transferred cells offer a promising noninvasive approach to studying their PK profile in vivo. Various radiolabeling methods have been developed, such as ^125^I‐PKH95,^15^ [^99m^Tc]hexamethylpropyleneamine oxime (HMPAO),^16^ ^51^Cr,^17^ ^111^In‐oxine,^18^ ^89^Zr‐oxine.^19^, ^20^ Among them, ^111^In‐oxine and ^99m^Tc‐HMPAO have been approved by the United States Food and Drug Administration to label white blood cells in the United States.^18^ Although both positron emission tomography (PET) and single photon emission computed tomography (SPECT) have moderate spatial resolution compared with other medical imaging techniques, the spatial resolution of current clinical PET scanners (2−5 mm) is higher than that of SPECT scanners (7−15 mm).^21^, ^22^ In addition, the recent development of whole‐body PET provides potential versatility and capabilities for this technology.^23^ 8‐Hydroxyquinoline (oxine) is a metal‐chelating ligand that can bind various metals via the pyridyl nitrogen and the hydroxyl group, which becomes deprotonated, allowing the formation of neutral, lipophilic metal complexes.^24^ In recent years, ^89^Zr‐(oxinate)_4_ has been widely used for long‐term tracking of cells for PET imaging due to the long half‐life (78.4 h) of radionuclide ^89^Zr.^25^, ^26^, ^27^ ^68^Ga‐(oxinate)_3_, based on the short half‐life (68 min) radionuclide ^68^Ga, has also been developed for short‐term tracking of cells.^28^ In addition to this, ^52^Mn(oxinate)_2_ and ^64^Cu(oxinate)_2_ have also been used for direct cell labeling.^29^, ^30^ Although it can only be tracked for a few hours, it can significantly reduce the absorbed dose during the imaging process. The main advantages of these radiometal‐oxine‐based radiolabeling and nuclear imaging methods for transplanted cells are the simplicity of the labeling procedure and the versatility. Furthermore, compared with indirect cell labeling, it does not require genetic modification as required for direct cell labeling, presenting a lower regulatory obstacle for clinical application. However, a major drawback of this methodology is that radionuclides within cells can leak out during metabolism and cell death,^31^, ^32^ making it challenging to distinguish between living cells, damaged cells, radioactive cell debris, or leaking radionuclides.^20^ This may affect the accuracy of tracking results, especially in tissues and organs where free radionuclides tend to accumulate. Therefore, the accurate in vivo distribution of the labeled cells must be ensured by removing the free radionuclides that may accumulate in tissues and organs.

Iron chelators, such as deferoxamine (DFO), deferasirox (DFS), and deferiprone (DFP), have gained significant clinical use for treating patients with iron overload disorders, such as hereditary hemochromatosis.^33^, ^34^, ^35^, ^36^ These chelators possess the ability to bind free iron ions or remove iron from proteins containing iron in the body, thereby facilitating their rapid excretion through the liver, kidneys, and gut.^37^ In addition, these chelators have demonstrated a good safety profile with no serious side effects despite being taken over long term.^38^, ^39^ In addition to its clinical use as an iron chelator, DFO's strong affinity for Zr^4+^ has led to its use for radiolabeling proteins and antibodies with ^89^Zr for PET imaging.^40^, ^41^ For example, DFO‐conjugated trastuzumab has been widely investigated as an ^89^Zr‐immunoPET tracer for HER2‐positive breast cancer detection.^42^ Our study was inspired by the remarkable ability of these iron chelators to eliminate free radiometal ions, such as ^89^Zr and ^68^Ga, in the body. We explored the efficacy of three iron chelators, DFO, DFS, and DFP, in removing free ^89^Zr and ^68^Ga ions in vivo using PET imaging and ex vivo biodistribution. Additionally, we determined the most effective iron chelator for the corresponding radiometals. Furthermore, we evaluated the PET imaging tracking of ^89^Zr‐(oxinate)_4_ and ^68^Ga‐(oxinate)_3_ labeled cells treated with iron chelators, and compared it with the original untreated approach.

## 2 RESULTS

### 2.1 Iron chelators remove extracellular but not intracellular radionuclides in viable cells

**FIGURE 1:**
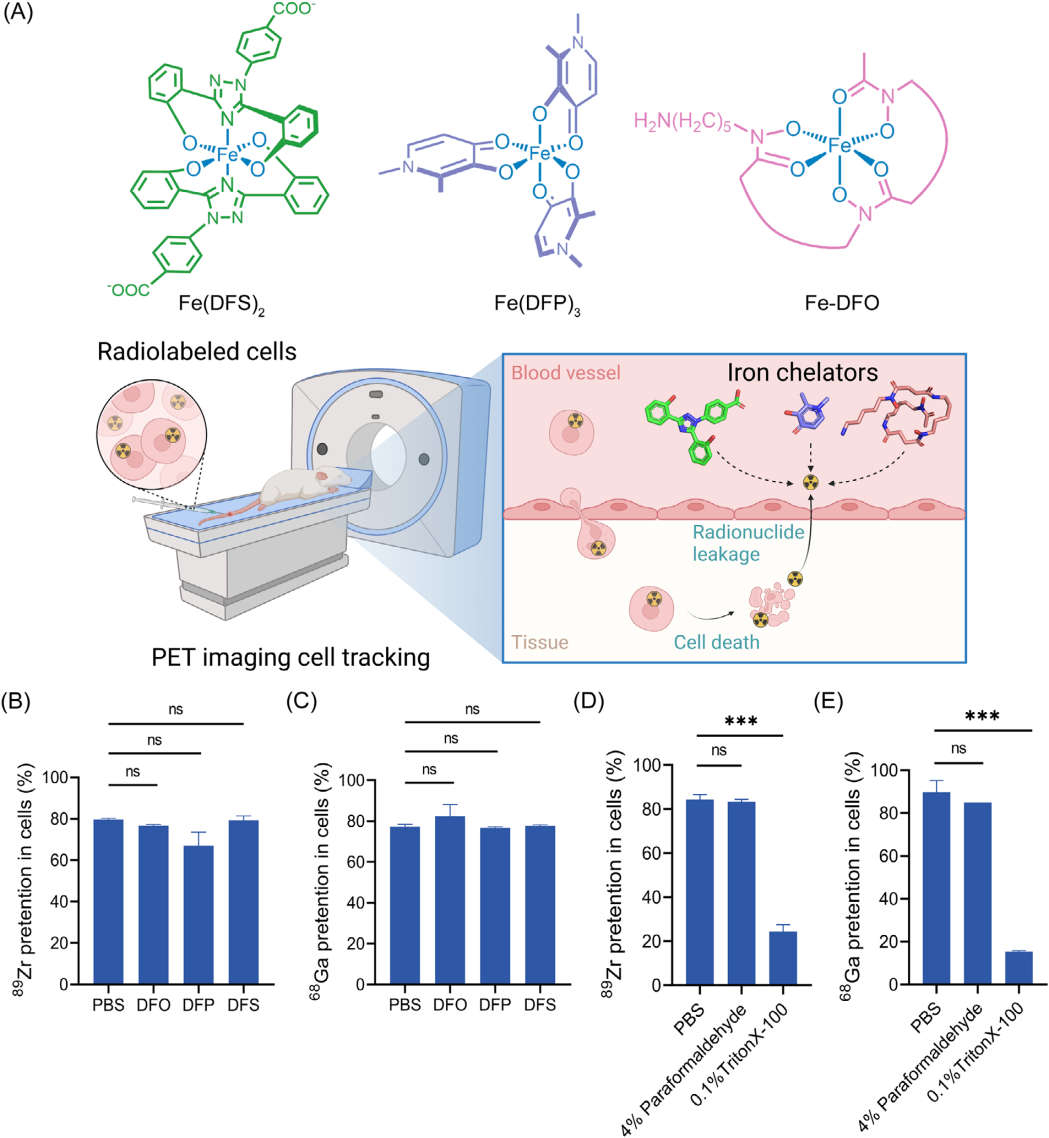
Iron chelators for in vivo removal of metal radionuclide leaked from radiolabeled cell therapies and in vitro radionuclide leakage evaluation. (A) Chemical structure of the chelators‐iron‐chelating complexes (Fe(DFS)_2_, Fe(DFP)_3_, and Fe‐DFO) and the schematic illustration of the free metal radionuclide removal from the radiolabeled transplanted cells in vivo for accurate PET imaging tracking. Created in biorender.com. (B) In vitro ^89^Zr retention in MSCs with various iron chelators treatment. (C) In vitro ^68^Ga retention in MSCs with various iron chelators treatment. (D) In vitro ^89^Zr retention and (E) ^68^Ga retention in MSCs with 4% paraformaldehyde, or 0.1% Triton X‐100 treatment. Values are expressed as the means ± SD (*n* = 3). ***p < 0.001, ns represents nonsignificant.

The study focuses on the removal of free metal radionuclides using iron chelators from in vivo radiolabeled transplanted cells for accurate PET imaging tracking. The chemical structure of three clinical used types of iron chelators is depicted in Figure 1A. In vitro experiments were conducted to determine if iron chelators remove radionuclides from viable radiolabeled cells, which could potentially affect the accuracy of the results. As depicted in Figures 1B and C, live MSCs maintained approximately 80% retention of ^89^Zr and ^68^Ga, with no significant differences from the PBS control. However, retention was markedly reduced by 0.1% Triton X‐100 treatment which disrupts membrane integrity, while paraformaldehyde fixation had little effect. (Figures 1D and E). In summary, these findings indicated that iron chelators remove extracellular but not intracellular radionuclides in viable cells.

### 2.2 Clearance of free ^89^Zr ions in vivo by iron chelators

**FIGURE 2:**
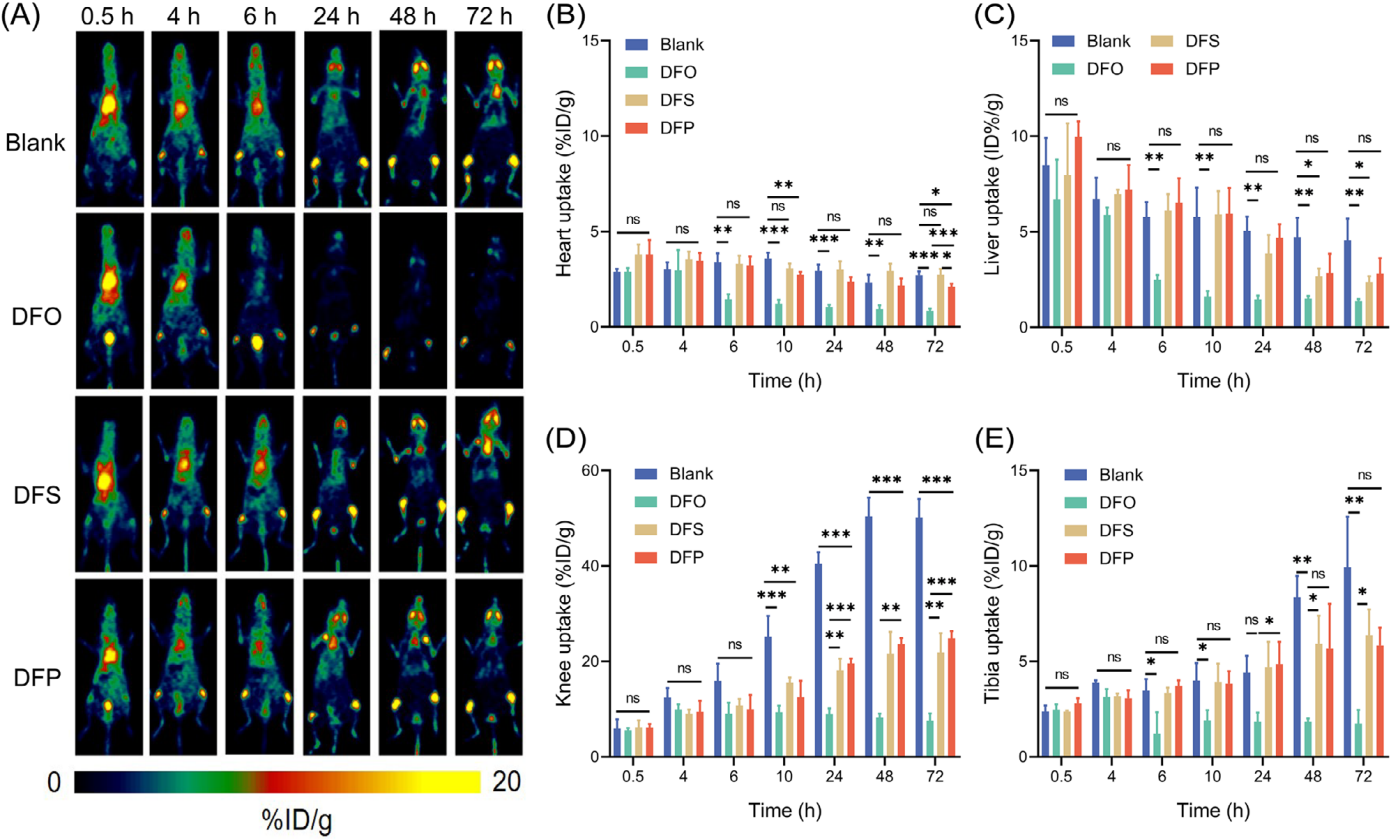
Evaluating the efficacy of iron chelators on free ^89^Zr ions removal in vivo. (A) Representative PET images of BALB/c mice injected with free ^89^Zr ions within 72 h. The mice treated with iron chelators DFO, DFS, and DFP were compared with a blank control group. Time‐course organ and tissue uptake of the free ^89^Zr ions based on the PET imaging in heart (B), liver (C), knee (D), and tibia (E). Values are expressed as the means ± SD (*n* = 3). *p < 0.05, **p < 0.01, ***p < 0.001, ns represents nonsignificant.

We embarked upon a study to investigate the efficacy of three iron chelators in clearing the free metal radionuclide ^89^Zr in vivo. To test the efficacy of the three iron chelators, mice were administered DFO intramuscularly or DFS and DFP orally prior to radionuclide injection. The doses and routes of administration aligned with standard clinical protocols for each chelator.^43^ DFO needs to be administered intramuscularly mainly due to its low oral bioavailability.^44^ Thereafter, intravenous injection of the free metal radionuclide ^89^Zr was carried out, followed by PET imaging. As depicted in Figure 2A, the free metal radionuclide ^89^Zr initially disseminated in the blood and liver after intravenous injection and subsequently settled in the bones, such as the knee and tibia, in line with extant literature reports.^45^

Tissue uptake was analyzed from the PET images, including heart, liver, knee, and tibia (Figures 2B–E). In the first 4 h, mice treated with iron chelators exhibited similar results to the blank control group, but substantial differences were observed among the groups commencing from the 6‐h time point postinjection. Of note, all three chelators significantly curtailed the uptake of free ^89^Zr in bones. At 72 h postinjection, the knee joint uptake values of the DFO, DFP, and DFS treatment groups were 7.63 ± 1.34, 24.83 ± 1.23, and 21.83 ± 3.34%ID/g, respectively, which were substantially lower than the value of 50.17 ± 3.15%ID/g in the untreated group. Specifically, the bone uptake in the DFO treatment group commenced a decline at 6 h postinjection. Furthermore, the uptake value in the liver plummeted to 2.48 ± 0.2%ID/g at 6 h postinjection, which was lower than the uptake value of 5.78 ± 0.63%ID/g in the control group, and even lower than the control group at 72 h postinjection. These data demonstrated that iron chelators can effectively eliminate free ^89^Zr in the body, resulting in a significant reduction in bone uptake, with DFO displaying the most robust clearance ability.

### 2.3 Clearance of free ^68^Ga ions in vivo by iron chelators

**FIGURE 3:**
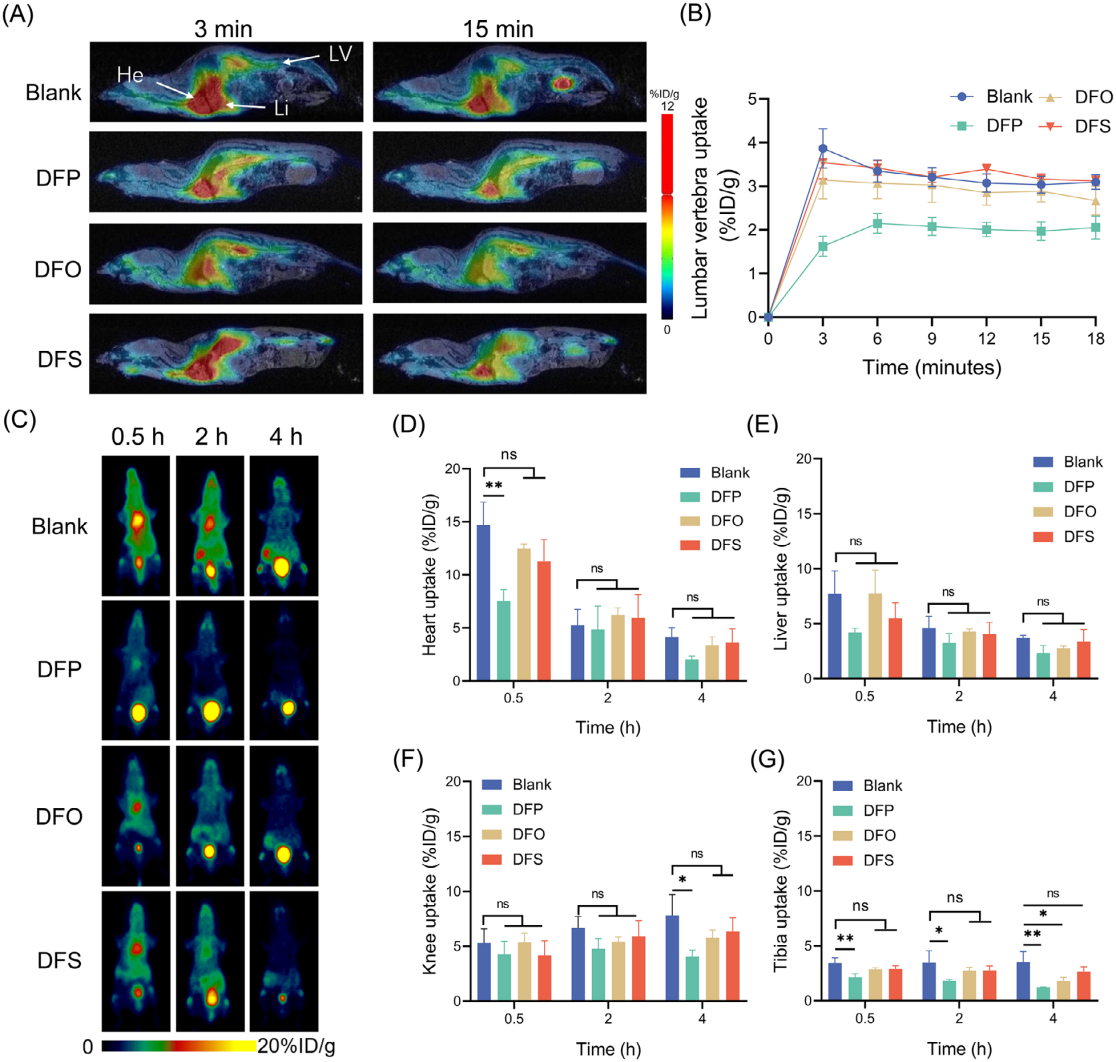
Evaluating the efficacy of iron chelators on free ^68^Ga ions removal in vivo. (A) PET/MR images of BALB/c mice injected with free ^68^Ga ions at 3 and 15 min postinjection. Organs and tissues were labeled, such as liver (Li), heart (He), and lumbar vertebrae (LV). (B) Lumbar vertebra uptake of the free ^68^Ga ions based on the PET imaging. (C) Representative PET images of BALB/c mice injected with free ^68^Ga ions within 4 h. The mice treated with iron chelators DFO, DFP, and DFS were compared with a blank control group. Time‐course organ and tissue uptake of the free ^68^Ga ions based on the PET imaging in heart (D), liver (E), knee (F), and tibia (G). Values are expressed as the means ± SD (*n* = 3). *p < 0.05, **p < 0.01, ns represents nonsignificant.

The short‐lived radionuclide ^68^Ga enables cell tracking over transient time frames.^30^ We compared the in vivo clearance capacities of ^68^Ga among iron chelators using PET imaging within 4 h and dynamic PET/MR imaging within 18 min. PET/MR images in Figures 3A and B revealed distinct in vivo distribution characteristics for the DFP treatment group compared with blank control, DFO and DFS cohorts shortly after ^68^Ga injection. Notably, bone deposition was substantially reduced with DFP from the earliest scan, unlike high accumulation seen in the untreated group. As shown in Figure 3C, the in vivo distribution of free Ga ions has similarities to that of free ^89^Zr within 4 h. Upon entering the blood circulation, ^68^Ga ions similarly accumulate in the bone. At the 0.5‐h time point, all four treatments show higher heart uptake compared with liver and knee uptake. Among the four treatments, blank control shows the highest heart uptake at 14.69 ± 2.17%ID/g, followed by DFO at 12.48 ± 0.43%ID/g, and DFS at 11.28 ± 2.05%ID/g, and DFP at 7.54 ± 1.08%ID/g (Figures 3D–G). At the 2‐h time point, all treatments show a decrease in heart, liver, and knee uptake compared with the 0.5‐h time point. A further decrease was found at the 4‐h time point. It is worth noting that both the PET images and uptake value showed that DFP‐treated mice have a remarkably low accumulation of ^68^Ga ions among all groups. The liver uptake decreased to 2.34 ± 0.68%ID/g and the knee uptake decreased to 4.06 ± 0.58%ID/g at the 4‐h time point.

To gain further insights into the competitive interactions of the three chelators with Ga ions and Zr ions, we conducted an ESI MS analysis. Intriguingly, our observations showed that the Ga‐DFP complex displayed the highest abundance when competing with Ga ions, while the Zr–DFO complex exhibited the highest abundance when competing with Zr ions (Figures S1 and S2). Overall, the results suggest that DFP treatment showed the strongest capability in reducing initial uptake and faster clearance of the free ^68^Ga ions in vivo.

### 2.4 In vivo PET imaging tracking of ^89^Zr‐labeled MSCs improved by DFO treatment

**FIGURE 4:**
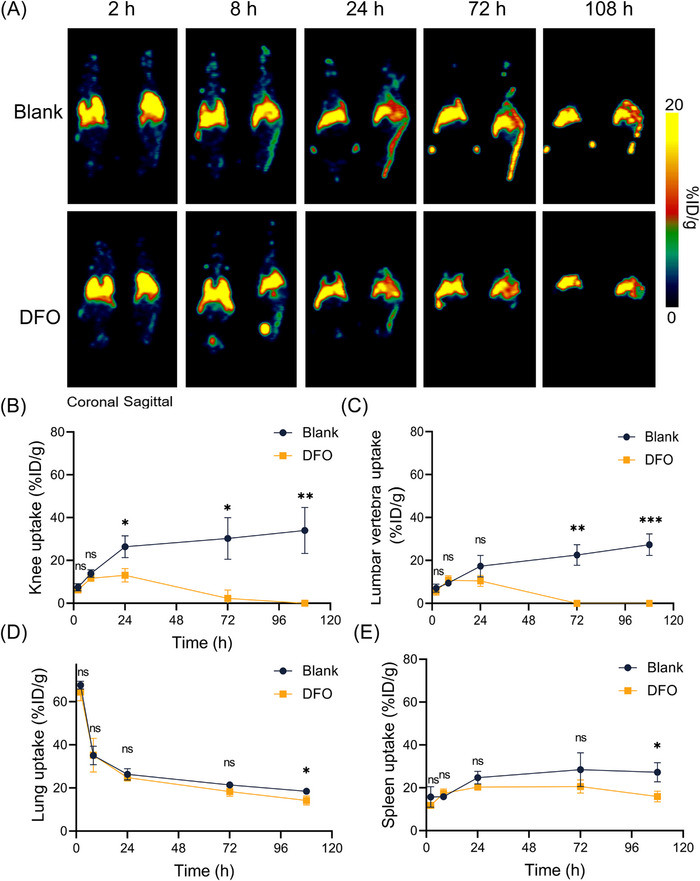
Iron chelator treatment improves PET imaging tracking of ^89^Zr‐labeled MSCs. (A) Representative PET images of intravenously transplanted ^89^Zr‐labeled MSCs in C57BL/6 mice within 108 h. The mice treated with iron chelators DFO were compared with a blank control group. Tissue uptake of ^89^Zr‐labeled MSCs in knee (B), lumbar vertebrae (C), lung (D), and spleen (E). Values are expressed as the means ± SD (*n* = 3). *p < 0.05, **p < 0.01, ***p < 0.001, ns represents nonsignificant.

In order to investigate the impact of these chelators on the PET imaging tracking of ^89^Zr‐labeled MSCs transplants, we employed the optimal iron chelator, DFO, to clear free ^89^Zr during the imaging process. The coronal and sagittal PET images obtained during the 108‐h tracking of the ^89^Zr‐labeled MSCs transplants (Figure 4A) indicate that the initial distribution of the transplants is predominantly in the lungs, followed by migration into other organs such as the liver and spleen. This trend is similar in both the groups with and without DFO treatment. However, the difference between the two groups lies primarily in the uptake in the bone, including the knee and lumbar vertebrae. Over time, bone uptake in the group without DFO treatment increases while it gradually decreases in the DFO‐treated group after a brief increase in the first 24 h. By 72 h postinjection and beyond, bone uptake was largely imperceptible on the PET images.

As shown in Figures 4B–E, the uptake values of the region of interest based on the PET images confirm this observation. The uptake of ^89^Zr‐labeled MSCs is very similar in the lungs in both the DFO‐treated and blank control groups, with some decrease in uptake values in the spleen and substantial differences in bones such as joints and lumbar vertebrae. At 108 h postinjection, the knee and lumbar vertebrae uptake in the blank control group were 34.03 ± 10.74 and 27.37 ± 4.97%ID/g, respectively, while in the DFO‐treated group, the uptake value decreased to almost zero. These findings demonstrate that DFO treatment can effectively remove the image disturbance caused by free ^89^Zr leaked from ^89^Zr‐labeled MSCs in vivo, particularly in the bone where ^89^Zr tends to accumulate.

### 2.5 Ex vivo biodistribution of ^89^Zr‐labeled MSCs

**FIGURE 5:**
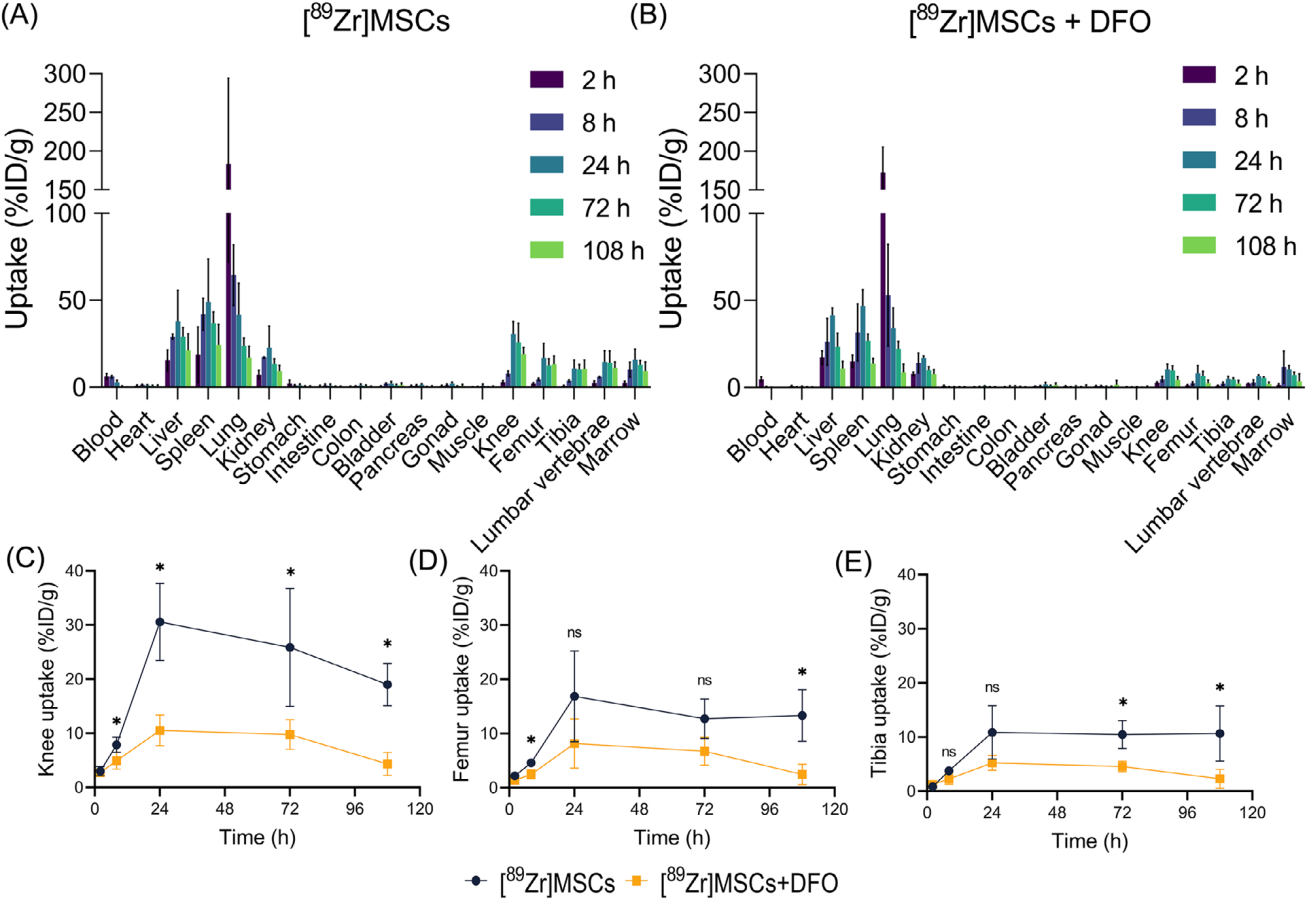
Iron chelator treatment decreases specific organ uptake of ^89^Zr‐labeled MSCs. Ex vivo biodistribution of intravenously transplanted ^89^Zr‐labeled MSCs in C57/BL6 mice at different time points (2, 8, 24, 72, and 108 h) without (A) or with (B) DFO treatment, respectively. Multiple organs and tissues were collected and measured, including blood, heart, liver, spleen, lung, kidney, stomach, intestine, colon, bladder, pancreas, gonad, muscle, knee, femur, tibia, lumbar vertebrae, and marrow. Enlarged chart for tissue uptake of ^89^Zr‐labeled MSCs in knee (C), femur (D), and tibia (E). Values are expressed as the means ± SD (*n* ≥ 3). *p < 0.05, **p < 0.01, ***p < 0.001, ns represents nonsignificant.

To comprehensively characterize the biodistribution of the ^89^Zr‐labeled MSCs, both DFO‐treated and untreated mice were intravenously administered with the radiolabeled cells, and their organs were taken out at 2, 8, 24, 72, and 108 h postinjection for radioactivity measurement. As shown in Figures 5A and B, The distribution of radioactivity differed between the two groups. Notably, the DFO‐treated group exhibited lower levels of radioactivity in most organs, indicating that the treatment was successful in reducing the amount of unbound ^89^Zr. Upon analyzing individual organs, the lungs, liver, spleen, kidney, bones, and marrow exhibited high levels of radioactivity in both groups, regardless of DFO treatment. Of note, the highest uptake occurred in the lungs at 2 h postinjection, with values of 228.74 ± 77.85 and 172.83 ± 32.68%ID/g in the untreated and DFO‐treated groups, respectively. Subsequently, the uptake values gradually decreased, in agreement with the PET imaging results. However, the liver, spleen, and kidneys showed a trend of increasing and then decreasing uptake, with the highest uptake occurring at 24 or 48 h postinjection.

One of the most significant differences in distribution between the two groups was observed in the bones, including the knee, femur, and tibia (Figures 5C–E). In the blank control group, bone uptake values increased over time and remained consistently high. In contrast, in the DFO‐treated group, bone uptake values initially increased slightly and then decreased, remaining low overall. Importantly, the bone marrow uptake in the DFO‐treated group increased from 1.61 ± 0.51 to 11.81 ± 9.17%ID/g between 2 and 8 h postinjection, while the lumbar vertebrae uptake only increased from 2.32 ± 0.31 to 3.14 ± 1.43%ID/g during the same period.

Based on ex vivo biodistribution of ^89^Zr‐labeled MSCs in mice with or without DFO treatment, the estimations of the human absorbed doses (mSv/MBq) to normal tissues are performed and summarized in Table 1. The results showed that the additional administration of DFO leads to a reduction in the absorbed doses for all the target organs and effective doses. Remarkably, the effective dose of ^89^Zr‐MSCs was reduced by nearly 34%, dropping from 0.201 to 0.132 mSv/MBq. Overall, the results suggest that additional administration of DFO when tracking ^89^Zr‐labeled MSCs leads to lower absorbed doses for all the target organs and effective dose.

**TABLE 1:** Human estimated equivalent doses and effective dose from ^89^Zr‐labeled MSCs cells with or without DFO treatment based on biodistribution in mice (Olinda).

	Dose estimate (mSv/MBq)	
Target organ	^89^Zr‐MSCs	^89^Zr‐MSCs+DFO
Adrenals	2.57E−01	1.68E−01
Brain	2.52E−01	1.65E−01
Esophagus	2.48E−01	1.62E−01
Eyes	2.52E−01	1.64E−01
Gallbladder wall	3.03E−01	1.98E−01
Left colon	3.29E−01	2.15E−01
Small intestine	3.50E−01	2.29E−01
Stomach wall	2.62E−01	1.71E−01
Right colon	3.55E−01	2.32E−01
Rectum	3.71E−01	2.42E−01
Heart wall	1.52E−01	9.95E−02
Kidneys	1.94E−01	1.27E−01
Liver	1.45E−01	9.48E−02
Lungs	1.72E−01	1.12E−01
Pancreas	2.09E−01	1.37E−01
Prostate	3.76E−01	2.45E−01
Salivary glands	3.22E−01	2.10E−01
Red marrow	2.43E−01	1.59E−01
Osteogenic cells	2.81E−01	1.84E−01
Spleen	2.09E−01	1.37E−01
Testes	2.20E−01	1.44E−01
Thymus	2.65E−01	1.73E−01
Thyroid	3.01E−01	1.97E−01
Urinary bladder wall	3.43E−01	2.24E−01
Effective dose	2.01E−01	1.32E−01

### 2.6 PK analysis of ^89^Zr‐labeled MSCs improved by DFO treatment

**FIGURE 6:**
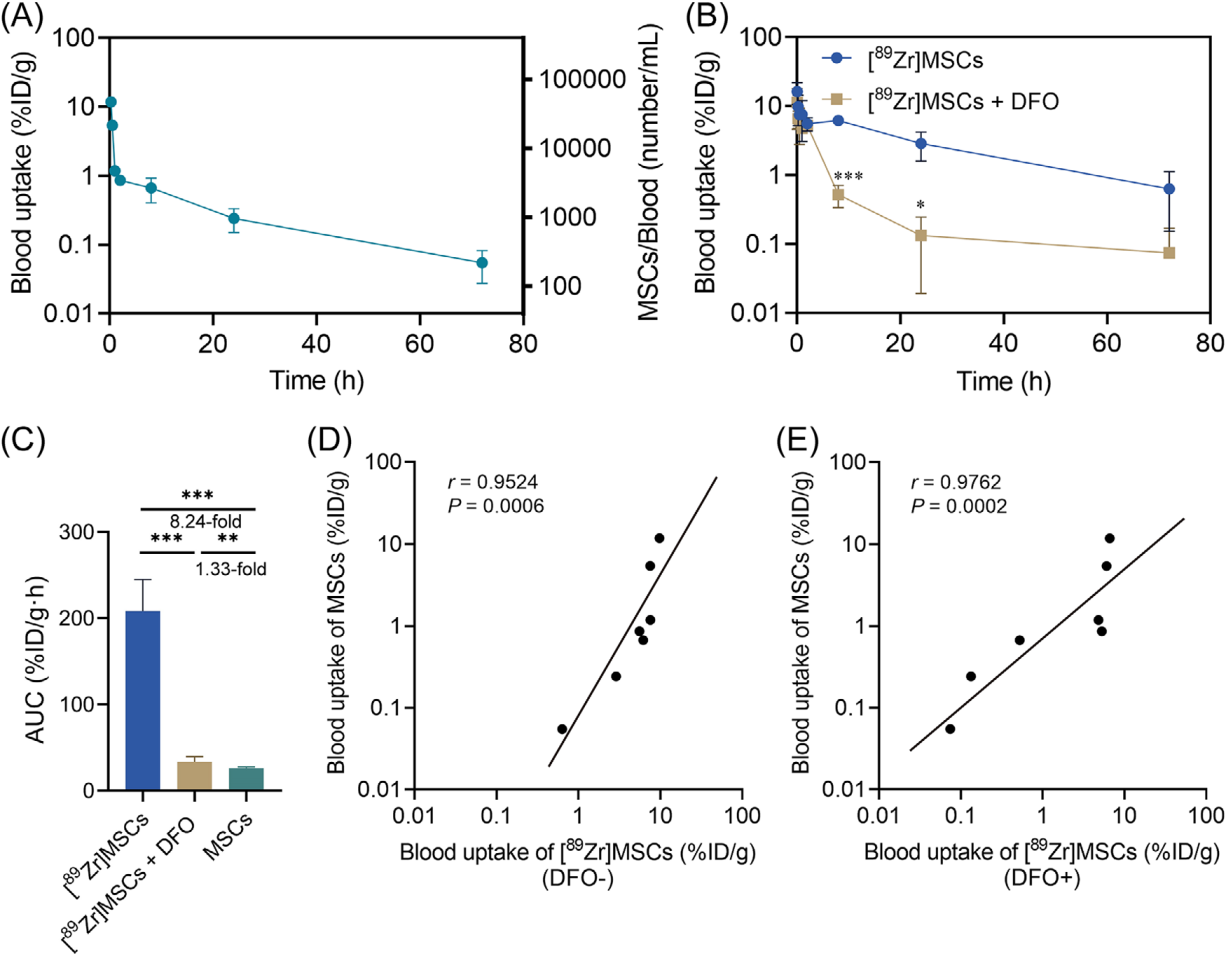
Iron chelator treatment improves blood pharmacokinetics analysis accuracy of ^89^Zr‐labeled MSCs. (A) Pharmacokinetics of intravenously transplanted MSCs in BALB/c mice. Mice were injected with 8 × 10^5^ MSCs and blood samples were collected at various time points. The numbers of human MSCs were quantified by quantitative real time‐polymerase chain reaction (qPCR) of human Alu (hAlu). (B) Pharmacokinetics of intravenously transplanted ^89^Zr‐labeled MSCs ([^89^Zr]MSCs) with or without DFO treatment. Mice were injected with 8×10^5^ MSCs with radioactivity of 185−190 KBq. (C) AUC(0−*t*) for the blood uptake of [^89^Zr]MSCs with or without DFO treatment, compared with the blood uptake of MSCs obtained by qPCR. (D) The correlation analysis was based on the blood uptake of [^89^Zr]MSCs without DFO treatment and blood uptake of MSCs obtained by qPCR. (E) The correlation analysis was based on the blood uptake of [^89^Zr]MSCs with DFO treatment and blood uptake of MSCs obtained by qPCR. Values are expressed as the means ± SD (*n* = 3). *p < 0.05, **p < 0.01, ***p < 0.001, ns represents nonsignificant.

We compared the γ‐counting method with qPCR methods for blood PK. Using human‐specific hAlu as a detection marker, we quantified human MSC numbers in samples by referencing a standard curve of hAlu cycle threshold (Ct) value in qPCR assays generated using known numbers of MSCs (Figure S3).^13^ We determined the PK profiles of intravenously transplanted MSCs using qPCR, with concentration shown as MSCs/blood (number/mL) and converted to blood uptake (%ID/g) by setting the blood weight of the mice. The concentration of MSCs in blood decreased rapidly from 47,193 to 4720 number/mL between 0.25 and 1 h postinjection, and then slowly declined over the following tens of hours (Figure 6A).

To determine the PK profiles of ^89^Zr‐labeled MSCs, we used γ‐counting. As shown in Figure 6B, there was a significant difference in tracking results between groups with or without DFO treatment. This difference occurred mainly at the 8‐h time point and beyond. At 8 h post‐MSC injection, the DFO‐treated mice had an MSC blood uptake value of 0.52%ID/g compared with 6.20%ID/g for untreated mice. At 24 h post‐MSC injection, the DFO‐treated mice had an MSC blood uptake value of 0.13%ID/g compared with 2.88%ID/g for untreated mice. This resulted in a substantial difference in the area under curve (AUC) between the two groups. The AUC of the untreated group was 208.20%ID/g h, while that of the DFO‐treated group was only 33.66%ID/g h, which was 8.24 and 1.33 times higher than the AUC obtained by the qPCR method, respectively (Figure 6C). We conducted a Pearson correlation analysis between the concentration determined by qPCR and γ‐counting in the untreated and DFO‐treated groups (Figures 6D and E). We found a significant correlation in both groups (*r* = 0.9524, *p* = 0.0006 for untreated group; *r* = 0.9762, *p* = 0.0002 for DFO‐treated group). These results suggest that DFO treatment can significantly improve the accuracy of γ‐counting as a blood PK assay, providing the basis for a noninvasive clinical method (PET imaging) to determine the blood PK of cell therapeutics.

### 2.7 PET imaging tracking of transplanted ^89^Zr‐labeled and ^68^Ga‐labeled bone marrow cells

**FIGURE 7:**
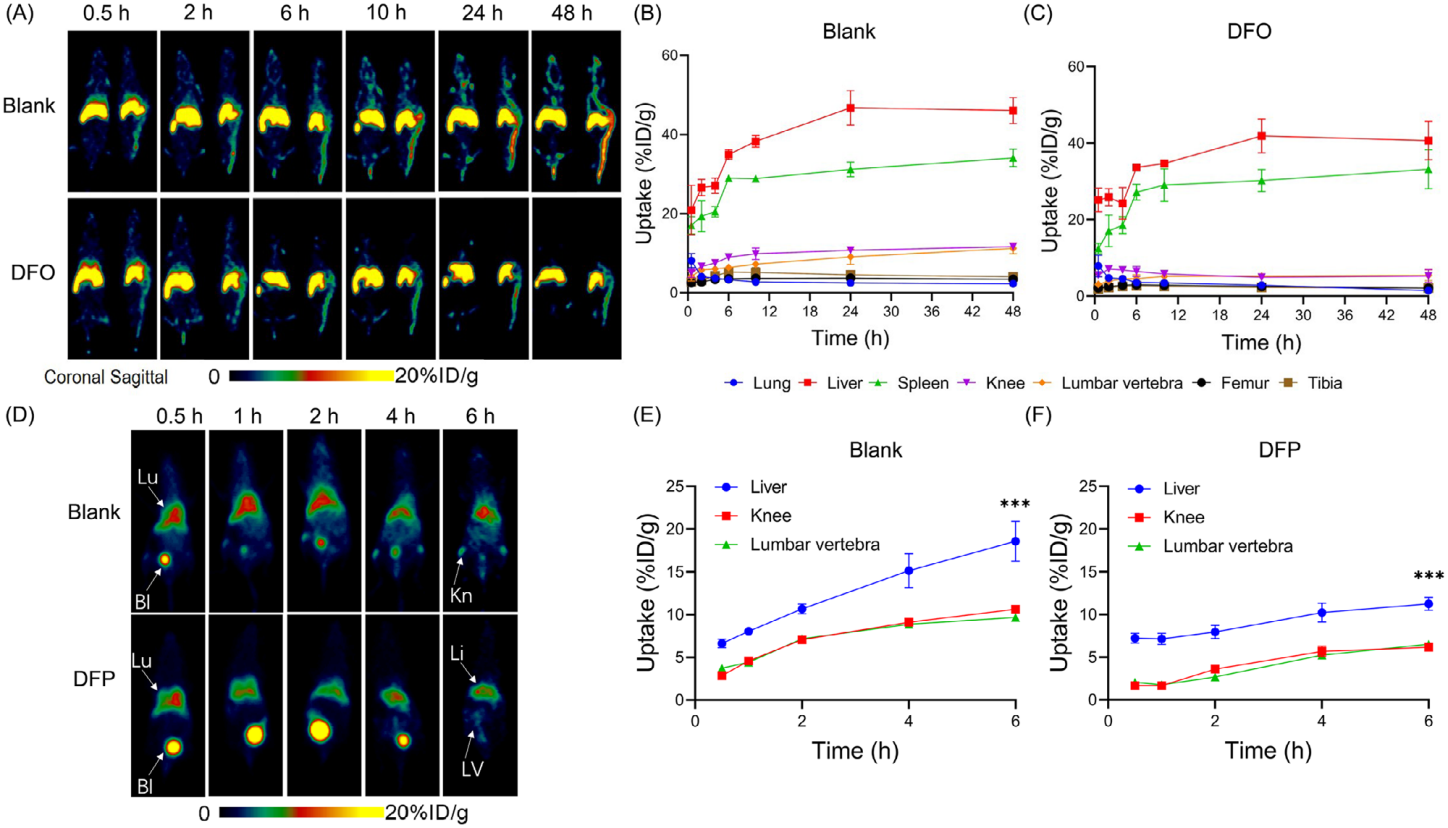
Iron chelator treatment improves PET imaging tracking of ^89^Zr‐labeled and ^68^Ga‐labeled bone marrow cells. (A) Representative PET images of intravenously transplanted ^89^Zr‐labeled bone marrow cells in C57BL/6 mice within 48 h. The mice treated with iron chelators DFO were compared with a blank control group. Tissue uptake of ^89^Zr‐labeled MSCs in lung, liver, spleen, knee, lumbar vertebrae, femur, and tibia for blank control group (B) and DFO‐treated group (C). (D) Representative PET images of intravenously transplanted ^68^Ga‐labeled bone marrow cells in C57BL/6 mice within 6 h. The mice treated with iron chelators DFP were compared with a blank control group. Organs and tissues were labeled, such as lung (Lu), liver (Li), bladder (Bl), Knee (Kn), and lumbar vertebrae (LV). Tissue uptake of ^68^Ga‐labeled MSCs in liver, knee, and lumbar vertebrae for blank control group (E) and DFP‐treated group (F). Values are expressed as the means ± SD (*n* = 3). ***p < 0.001, ns represents nonsignificant.

To evaluate the potential of DFO treatment to enhance PET imaging tracking of the homing process after bone marrow transplantation, we performed PET imaging tracking after labeling bone marrow cells from mice with ^89^Zr‐oxine or ^68^Ga‐oxine and infused them back into the mice. The successful radiolabeling of bone marrow cells with ^89^Zr‐oxine or ^68^Ga‐oxine was confirmed by radio‐TLC (Figure S4). The in vivo distribution of ^89^Zr‐labeled bone marrow cells was quite different from that of ^89^Zr‐labeled MSCs, as shown in Figures 7A and 4. ^89^Zr‐labeled MSCs had high lung uptake in the initial few hours of infusion, while bone marrow cells did not. Bone uptake was substantially lower in the DFO‐treated mice than in the blank control. The images revealed high uptake in the spine but no significant uptake in the scapula and knee joints. Furthermore, after 48 h, the ^89^Zr‐labeled MSCs were removed from the spine with a very low level, whereas the bone marrow cells remained. Despite higher uptake values in the spine in the blank control group, nonspecific bone uptake clearly interfered with bone marrow homing process observation. The veracity of this observation can be further corroborated by examining the uptake values presented in Figures 7B and C. Notably, in the blank control group, the uptake values at 48 h postinjection in the lumbar vertebrae and tibia stand at an impressive 11.23 ± 0.35 and 4.03 ± 0.60%ID/g, respectively. Conversely, in the DFO‐treated group, these uptake values demonstrate a marked reduction, measuring at 5.47 ± 1.42 and 2.13 ± 0.51%ID/g for the knee and lumbar vertebrae, respectively.

We also labeled bone marrow cells with ^68^Ga to track the in vivo distribution homing process. The distribution of ^68^Ga‐labeled bone marrow cells was mainly observed in the liver and spleen, similar to that of ^89^Zr‐labeled bone marrow cells, as shown in Figures 7D–F. DFP treatment significantly reduced uptake in bone. Liver uptake was also substantially reduced after DFP treatment. Knee and abdominal uptake were evident in the blank control group, whereas lumbar region uptake was observed in the DFP‐treated group. These findings demonstrate that DFO treatment improves the accuracy of PET imaging in monitoring the success and persistence of bone marrow transplantation.

### 2.8 Activity evaluation of ^89^Zr‐ and ^68^Ga‐labled MSCs

To ensure that the radiolabeling process within the specific activity range detailed in this study had no adverse impact on the activity or function of MSCs, we meticulously conducted multiple assays focusing on viability, proliferation capacity, marker gene levels, and stem cell senescence. Remarkably, the viability of the MSCs remained virtually indistinguishable from that of their unlabeled counterparts for up to 120 h after the radiolabeling procedure, regardless of whether ^68^Ga labeling or ^89^Zr labeling was employed (Figure S5). Within a shorter timeframe of 72 h, we also found that their proliferative capacity remained entirely unaffected (Figure S6). Furthermore, we thoroughly examined the marker gene expression and senescence level of the MSCs and discovered that neither aspect was adversely affected by the radiolabeling process (Figures S7 and S8). These compelling results provide conclusive evidence that radiolabeling MSCs within the specific activity range chosen for this study is entirely safe and does not significantly impact the MSCs' activity.

## 3 DISCUSSION

In recent decades, cell therapies have emerged as promising treatments due to their unique efficacy against certain diseases.^1^ However, evaluating the PK and in vivo distribution of these “living drugs” presents challenges compared with traditional chemical agents or antibody‐based biologics. Consequently, numerous cellular imaging technologies have been developed, including decades‐old radiometal‐labeling approaches such as ^111^In‐oxine and ^89^Zr‐oxine for direct cell radiolabeling.^18^, ^19^ While convenient and clinically translatable, a major limitation of direct labeling is the potential efflux of radionuclides from cells after administration, confounding analysis.^20^ This tracer efflux hinders precise tracking and PK assessments, restricting clinical implementation. Iron chelators are clinically utilized drugs for treating iron overload disorders like hereditary hemochromatosis.^34^ Capitalizing on the similarities between some radiometals and iron, we aimed to repurpose these chelators to address the issue of radionuclide efflux from administered cells in vivo. By leveraging their established ability to bind and remove free iron, we hypothesized iron chelators could selectively eliminate unbound radionuclides leaking from dead cells after infusion. This could improve PET imaging accuracy by removing background signals without affecting the detection of viable labeled cells. Our approach exploited the known iron‐clearing properties of chelators through drug repurposing in order to solve the clinical problem of radionuclide efflux interfering with cell tracking analysis.

Our in vitro findings demonstrate that iron chelators selectively remove extracellular, not intracellular, radionuclides in viable cells. Both ^89^Zr and ^68^Ga retention remained high in viable MSCs, unaffected by chelator treatment. However, membrane disruption with Triton X‐100 caused rapid radionuclide release, unlike paraformaldehyde which preserves membranes.^46^, ^47^ This implies iron chelators act solely on free extracellular radionuclides leaked from dead cells, without stripping radionuclides from living cells. These results underscore the utility of iron chelators for eliminating background signals in vivo without interfering with the detection of viable labeled cells.

Among the iron chelators, DFO exhibited the strongest in vivo clearance of free circulating ^89^Zr, likely attributed to its optimal binding affinity for Zr ions. With DFO treatment, ^89^Zr uptake in bones started declining by 6 h and was significantly reduced by 72 h. Bone tends to avidly bind Zr, making elimination challenging once deposited. Increased bladder uptake indicated renal clearance of DFO‐bound ^89^Zr. For ^68^Ga, DFP demonstrated superior rapid in vivo clearance over other chelators, attributable to its higher formation constant with Ga ions. As reported, the Ga formation constants (logβ) were found to be 32.6 and 27.6 for DFP and DFO,^36^ respectively, indicating DFP's superior affinity for Ga ions. DFP treatment minimized early ^68^Ga accumulation in heart, liver, and bone. The distinct chelator preferences between the two radionuclides underscore the significance of preferential metal binding in determining removal efficacy.

DFO treatment during ^89^Zr‐MSC tracking significantly reduced bone uptake without altering major organ distribution. This selective elimination of free ^89^Zr signal enhanced imaging accuracy by avoiding bone background interference that could mask the detection of viable cells. The biodistribution study further showed DFO decreased overall ^89^Zr levels while enabling observation of early marrow migration before bone deposition, consistent with previous reports in the literature.^48^ This highlights the value of selective free radionuclide removal in improving sensitivity for cell tracking and fate assessment. The substantial 34% decrease in estimated human radiation dose with DFO demonstrates the advantage of minimizing unbound radionuclides. Lower exposure benefits patients and enables higher cell administrations. Furthermore, we demonstrated that radiolabeling within the investigated activities did not adversely impact MSC viability, function, or phenotype. This attests to the safety of this approach for diverse cell‐tracking applications where maintaining cell integrity is paramount.

The γ‐counting/PCR correlation was stronger in the DFO group compared with untreated controls. This indicates that removing background ^89^Zr with DFO enhances the quantitation accuracy of PET for noninvasive cell PK analysis. Since PCR‐based methods require blood sampling and ex vivo analysis, having a noninvasive PET imaging approach that closely matches PCR quantification due to a reduced radionuclide background would be highly valuable clinically. Our findings demonstrate that PET tracking coupled with iron chelator treatment to clear extracellular radionuclides could potentially provide a widely accessible method for longitudinal monitoring of cell persistence and clearance kinetics in patients receiving cell therapies. This supports the utility of PET tracking for noninvasive cell PK assessment when background radionuclides are removed.

Bone marrow transplantation is a versatile therapy that can effectively treat a range of conditions, such as blood cancers, inherited blood disorders, autoimmune disorders, and tissue engineering.^49^, ^50^, ^51^ Noninvasive bioimaging techniques, such as PET, SPECT, and MR, can monitor the success and persistence of hematopoietic stem cell transplantation in bone marrow transplantation.^52^ For bone marrow transplant monitoring, DFO and DFP treatments increased PET imaging precision by preventing nonspecific bone accumulation that obscured homing signals. Avoiding free radionuclide deposition is key for accurate tracking of bone‐localized cells.

While direct biological studies are still needed for confirmation, the substantially reduced radiation doses with chelators would likely confer multiple benefits, including decreased DNA damage, reduced oxidative stress, preserved function in normal organs, and protection of radiosensitive blood cell populations.^53^, ^54^, ^55^ Thus, mitigating radiation exposure by removing free radionuclides with iron chelators may enable safer administration of higher therapeutic radiolabeled cell doses and repeat scanning.

We envision this strategy could broadly benefit PET cell tracking in therapeutic applications undergoing active translation like monitoring tumor‐localized CAR‐T cells and TILs in cancer immunotherapy, tracking biodistribution and evaluating homing/engraftment of MSCs and bone marrow transplantation in regenerative medicine, assessing islet graft survival after transplantation in diabetes patients.^1^, ^28^, ^32^, ^52^ More accurate quantification of cell persistence by avoiding radionuclide background could aid efficacy and safety evaluations.

While the iron chelators used in this study have established safety profiles, their clinical translation for PET imaging would still warrant careful consideration of risks like skin rashes, gastrointestinal upset, bone/joint pain, and in rare cases, agranulocytosis or ocular/auditory toxicity.^56^ Short‐term use would unlikely cause major side effects but precautions would be needed for certain populations and with repeated administrations. Contraindications such as renal impairment must also be considered. Overall, iron chelators pose low long‐term risks but their incorporation for cell tracking would require weighing the benefits of improved imaging accuracy against potential chelator‐associated adverse effects through rigorous risk‐benefit analyses. Further investigations of chelator PK, optimal dosing, and clinical safety profiles would strengthen the translational outlook of this approach.

In summary, our study has demonstrated that the optimized iron chelators, DFO and DFP, are highly effective in removing free radionuclides ^89^Zr and ^68^Ga in vivo, respectively. This approach enables the removal of free radionuclides released from dead cells during radiolabeled cell tracking without interfering with the signal from living cells, resulting in more accurate and reliable PET imaging tracing. When used for PET imaging tracking of ^89^Zr‐labeled MSCs, the use of DFO significantly reduces uptake in bone while preserving uptake in major organs such as the lung, liver, and spleen. The clearance of free ^89^Zr in vivo also results in a significant reduction in the absorbed dose of radiation from ^89^Zr‐labeled MSCs. Additionally, the avoidance of accumulation of free ^89^Zr and ^68^Ga into the bone allows for more precise observation of the homing process and persistence during bone marrow transplantation. Given the efficacy and safety of this solution, we believe it has the potential to be widely used for imaging tracking studies after direct labeling of various cells.

## 4 MATERIALS AND METHODS

### 4.1 Materials

8‐Hydroxyquinoline (oxine), sodium citrate, NaOAc, Na_2_CO_3_, and 2‐[4‐(2‐hydroxyethyl)‐piperazin‐1‐yl] ethane sulfonic acid (HEPES) solution were purchased from Sigma–Aldrich (St Louis, MO). Dimethyl sulfoxide (DMSO) was obtained from Acros Organics (Belgium, USA). DFS was purchased from Innochem (Beijing, China). DFP was purchased from MedChem Express Ltd. Deferoxamine mesylate (DFO) was purchased from Novartis China. Gallium nitrate hydrate and zirconium tetrachloride were obtained from Innochem (Beijing, China). ^89^Zr‐oxalate was obtained from Dongcheng AMS Pharmaceutical Co., Ltd (Nanjing, China). ^68^Ga^3+^ solution was eluted from a ^68^Ge/^68^Ga generator (ITG, Germany) with 4 mL of 0.05 M HCl (Merck, Germany), from which the 0.5 mL fraction with the highest radioactivity was used for further experiments. β‐Actin internal reference primers, MSCs markers primer sequences used for real‐time qPCR were obtained from Sangon Biotech Co., Ltd. (Shanghai, China).

### 4.2 Cell culture and animals

MSCs were obtained from Jiangsu Ruiyuan Biotechnology Co., Ltd (Jiangsu, China). The cells were cultured in DMEM/F12 (Gibco, Carlsbad, CA, USA) medium supplemented with 10% fetal bovine serum (Gibco, Carlsbad, CA, USA) and 1% penicillin−streptomycin (Beyotime Biotechnology, Shanghai, China) at 37°C in a humidified 5% CO_2_ atmosphere. The MSCs were cultured to passage 6 for subsequent experiments.

Bone marrow cells were obtained from 8 to 10 weeks‐old C57BL/6 mice. After the mice were sacrificed by neck dislocation and immersed in 70% (v/v) ethanol for 2 min. The hind limb muscles, femur, and tibia were carefully separated using ophthalmic scissors. The bone was then held with forceps, and the two ends were excised from the marrow cavity, which was rinsed with Hank's balanced salt solution until it became pale. The bone marrow was passed through a 70‐mm nylon cell strainer (Beyotime Biotechnology, Shanghai, China), and red blood cell lysate was added. The mixture was then centrifuged at 590 *g* for 5 min to harvest the bone marrow cells, which were resuspended in PBS buffer for subsequent experiments.

Adult female C57BL/6 and BALB/c mice (6−8 weeks old) were bought from CAVENS (Changzhou, China). All animals were maintained under specific‐pathogen‐free conditions with a constant temperature (23 ± 1.5°C) and humidity (70 ± 20%) on a 12‐h light/dark cycle.

### 4.3 Iron‐removing drugs remove free ^89^Zr and ^68^Ga from the body

DFO (50 mg/kg) was administered intramuscularly, while DFS (26 mg/kg) and DFP (26 mg/kg) were administered by gavage in healthy BALB/c mice (3 mice/group). Thirty minutes later, 0.37 MBq free ^89^Zr ions or 3.7 MBq free ^68^Ga ions were injected into the tail vein. The iron chelators were then administered three times daily.

### 4.4 Radiolabeling of MSCs and bone marrow cells

The radiolabeling was performed as reported in previous studies.^28^ For ^89^Zr‐oxine labeling, the pH of the ^89^Zr^4+^ solution was adjusted to seven using 1 M Na_2_CO_3_ and 0.1 M HEPES buffer. Then, 37 MBq ^89^Zr^4+^ solution was mixed with 10 µg oxine (5 µg/µL) dissolved in DMSO and allowed to react for 15 min at room temperature. For ^68^Ga‐oxine labeling, the pH of the ^68^Ga^3+^ solution was adjusted to 5−6 using 1 M NaOAc, and then the solution was mixed with 10 µg oxine (5 µg/µL) dissolved in DMSO and the reaction was allowed to proceed for 15 min at room temperature.

For radiolabeled cells, 1 × 10^6^ cells were incubated with 0.37 MBq ^68^Ga or ^89^Zr‐oxine solution at room temperature for 15 min and then centrifuged at 380 *g* for 4 min. Finally, the ^68^Ga and ^89^Zr‐labeled cells were washed three times with PBS buffer. The radioactivity of the cells and supernatant was measured using a radioactivity meter (PTW, Freiburg, Germany) to calculate the cell labeling efficiency. After labeling, the radiochemical purity of radiolabeled products was analyzed using radioactive thin‐layer chromatography (TLC, BioScan, USA). Radioactive transient thin‐layer chromatography was performed on thin‐layer chromatography glass fiber paper using 0.5 M citrate buffer solution (pH = 5) as the mobile phase. Intact radiolabeled cells were maintained at baseline while unbounded ^89^Zr^4+^ and ^68^Ga^3+^ ions migrated at the solvent front.

### 4.5 In vitro cell experiments

The ^89^Zr and ^68^Ga‐labeled cells were divided into 4 groups (*n* = 3): PBS, DFO/DFP, 4% paraformaldehyde, and 0.1% TritonX‐100. The radioactive dose of ^68^Ga and ^89^Zr added to each tube was 100 KBq. The different buffers were processed for 30 min, and then the cell suspension was passed through a 0.22 µm microporous membrane (Tansoole, Shanghai, China). The liquid was filtered into γ counting tubes, while the cell pellet was left in the filter membrane. The CPM of the γ counting tubes was measured, and the radioactive dose of the precipitation and supernatant was calculated.

### 4.6 Micro‐PET imaging

Static microPET scans were performed on an Inveon microPET scanner (Siemens Medical Solutions, Erlangen, Germany). Dynamic microPET/MRI scans were performed on a 9.4 T Bruker BioSpec 94/30 USR with PET insert (Bruker Biospin, Ettlingen, Germany). In order to monitor and compare the changes of PET images by iron removal drugs. C57BL/6 mice were intravenously injected with 5 × 10^689^Zr‐radiolabeled bone marrow cells, 8 × 10^589^Zr‐radiolabeled MSCs, and 5 × 10^668^Ga‐radiolabeled bone marrow cells via the tail vein. The radioactivity of each mouse was 180−185, 80−90, and 3.3–3.7 MBq, respectively. For ^68^Ga imaging, 10‐min‐long static PET scans were conducted at 2, 4, and 6 h p.i. For ^89^Zr imaging, 10 min‐long static PET scans were performed at 30 min, 2, 4, 8, 24, and 48 h p.i.

### 4.7 Micro‐PET imaging data processing and analysis

PET images were reconstructed using a three‐dimensional ordered subset expectation maximum algorithm. The static scan image data were analyzed using ASIPro software (Concorde Microsystems, Inc., TN, USA). The dynamic scan image data were analyzed using PMOD v.4.4 (PMOD Technologies Ltd., Zurich, Switzerland). The major organs and tissues, such as the lungs, liver, and spleen were delineated manually on the images as the regions of interest (ROIs). The image‐derived percentage injected dose per gram (%ID/g) was calculated for each ROI to quantify radioactivity uptake. The %ID/g can be obtained using the following equations:

$$\begin{array}{ccc} \%\mathrm{ID}/g & = & \mathrm{ROI}\;\mathrm{activity}\;\mathrm{concentration}\left(\mathrm{kBq}/\mathrm{mL}\right)/\\ & & \mathrm{Total}\;\mathrm{injected}\;\mathrm{activity}\left(\mathrm{kBq}\right)\times 100 \end{array}$$

### 4.8 Ex vivo biodistribution

C57BL/6 mice were intravenously injected with 8 × 10^589^Zr‐labeled MSCs. Then, the mice were sacrificed at 2, 8, 24, 72, and 108 h p.i. Samples of major organs and tissues, including the brain, liver, heart, spleen, lung, kidney, stomach, muscle, tibia, femur, and knee were removed and weighed. To isolate the knee joint, an incision was made to reveal the femur, tibia, and knee ligaments. The knee was then carefully excised by transecting the ligamentous and musculotendinous attachments to the distal femur and proximal tibia. The radioactivity of each sample was measured using a γ‐counter (2480 WIZARD2; PerkinElmer, USA), and then the %ID/g per organ was calculated. The biodistribution of the other experimental groups was also evaluated after PET imaging.

$$\begin{array}{ccc} \mathrm{Organ}\;\mathrm{uptake}\left(\%\mathrm{ID}/g\right) & = & \left(\mathrm{organ}\;\mathrm{cpm}\;\times \;\mathrm{standard}\right.\\ & & \left.\mathrm{dilution}\;\mathrm{rate}\;\times \;100\right)/\\ & & \left(\mathrm{standard}\;\mathrm{cpm}\times \mathrm{organ}\;\mathrm{weight}\right) \end{array}$$

### 4.9 PK study

The PK of ^89^Zr‐labeled MSCs were studied in C57BL/6 mice after intravenous injection of 8 × 10^589^Zr‐labeled MSCs, with a radioactivity of 185−190 kBq per mouse. Blood samples were collected from the tail at different times after intravenous injection and weighed. The radioactivity of the blood samples was measured using a γ‐counter and normalized to %ID/g. The AUC of blood uptake (%ID/g) versus time was calculated using the noncompartmental fitting method of DAS 2.0 software (Drug and Statistics 2.0; Mathematical Pharmacology Professional Committee of China, Shanghai, China).

### 4.10 Genomic DNA extraction and qPCR assay of hAlu

A micro genomic DNA extraction kit (DP316; TIANGEN BIOTECH BEIJING, CO., LTD, China) was used to extract genomic DNA (gDNA) from blood according to the manufacturer's protocol. In brief, approximately 20 µL of blood from the tail veins were added to tubes containing 100 µL of lysis buffer and 10 µL of proteinase K (20 mg/mL). The suspension was then digested in a water bath at 56°C. The gDNA was purified and eluted following the manufacturer's protocol.

Quantitative PCR primers were designed to target the human‐specific unique sequence of Alu and β‐actin. Primer sequences are shown in Table S1. Quantitative real‐time PCR assays were performed by Applied Biosystems ABI 7500 system (Thermo Fisher, Waltham, MA, USA). The reaction mixture was loaded into a PCR 8‐Tube, and qPCR assays were performed in a 20 µL volume containing 10 µL of qPCR SYBR Green mix (Yeasen Biotech, Shanghai, China), 0.4 µL of forward and reverse primers, and 20 ng of template gDNA diluted in water. SYBR Green PCR reaction conditions were as follows: the holding phase was 95°C for 2 min. Cycles were 40 cycles of 95°C for 10 s and 60°C for 34 s.

### 4.11 Cell number‐cycle threshold standard line establishment

Cells (1 × 10^6^, 2.5 × 10^5^, 6.25 × 10^4^, 3.125 × 10^4^, 7800, 2000, 500, 250, 125, 60) were mixed with 20 µL mice blood to extract gDNA. The standard curve of cycle threshold values versus cell number (logarithmic form) was obtained through qPCR amplification. The number of test blood mesenchymal MSCs was calculated from the standard curve.

### 4.12 Absorbed dose estimates

The activity and concentration data for various organs or tissues, including lung, liver, spleen, blood (left ventricular chamber), muscle, and other organs, were obtained by drawing ROIs on the PET images of C57BL/6 mice. The organ residence times of the mice were then fitted with PMOD v.3.8 (PMOD Technologies Ltd., Zurich, Switzerland). Using the calculation method outlined in the OLINDA software handbook, the residence time for each organ in the mouse was extrapolated based on the adult male (73 kg) reference. The remainder of the body residence time was determined based on the maximum theoretical residence time minus the sum of the residence times observed in the organs. The human dosimetry estimates were derived from the residence times using OLINDA/EXM 2.0 software (Hermes Medical Solutions, Stockholm, Sweden) with the adult male model.

### 4.13 Statistical analysis

GraphPad Prism 8.0 software (GraphPad Software, Boston, Massachusetts, USA) was used to perform statistical analysis on the data, which involved evaluating the significance of differences between groups. The data were presented as mean values and standard deviations, unless otherwise noted. The statistical significance of differences between groups was evaluated using one‐way ANOVA followed by Tukey's multiple comparisons test: *, *p* < 0.05; **, *p* < 0.01; ***, *p* < 0.001; ns, not significant, *p* > 0.05. The analyses were performed using replications derived from the same experiment.

## AUTHOR CONTRIBUTIONS

*Conceptualization*: X. W. and M. Y. *Investigation*: Q. X., Z. M., Y. Z., X. J., and X. D. *Project administration*: D. P., Y. X., and L. W. *Resources*: M. Y., J. W., and G. W. *Supervision*: X. W., M. Y., and G. W. *Visualization and writing—original draft*: Q. X. and X. W. *Writing—review and editing*: J. Y., C. C., X. W., and M. Y. All authors have read and approved the final manuscript.

## CONFLICT OF INTEREST STATEMENT

Author Jing Wang is a deputy director in Jiangsu Renocell Biotech Co., Ltd. The other authors have no conflicts of interest to declare.

## ETHICS STATEMENT

All animal experiments were approved by the Institutional Animal Care and Ethics Committee of Jiangsu Institute of Nuclear Medicine (Wuxi, China) (approval number: JSINM‐2023‐051).

## Supporting information

Supporting Information

## Data Availability

The data included in this study are available upon request from the corresponding author.
