# Supplementary material for: Repurposing iron chelators for accurate positron emission tomography imaging tracking of radiometal‐labeled cell transplants
Source: MedComm (2020). 2024 Jan 30;5(2):e473. doi: 10.1002/mco2.473 (PMC10827001; doi:10.1002/mco2.473)
Supplement: Supplementary file 1 — Supporting Information [file MCO2-5-e473-s001.docx]

**Supplementary Information**

# Repurposing iron chelators for accurate positron emission tomography imaging tracking of radiometal-labeled cell transplants

Qian Xu^1,2^, Xinyu Wang^1,2,^*, Ziqian Mu^1,2^, Yixiang Zhou^1,2^, Xiang Ding^1,2^, Xin Ji^2^, Junjie Yan^1,2^, Donghui Pan^2^, Chongyang Chen^2^, Yuping Xu^1,2^, Lizhen Wang^2^, Jing Wang^3^, Guangji Wang^4,^*, Min Yang^1,2,^*

^1^ Department of Radiopharmaceuticals, School of Pharmacy, Nanjing Medical University, Nanjing 211166, PR China;

^2^ NHC Key Laboratory of Nuclear Medicine, Jiangsu Key Laboratory of Molecular Nuclear Medicine, Jiangsu Institute of Nuclear Medicine, Wuxi 214063, PR China;

^3^ Jiangsu Renocell Biotech Co., Ltd., Nanjing 211100, PR China;

^4^ Key Laboratory of Drug Metabolism and Pharmacokinetics, State Key Laboratory of Natural Medicines, China Pharmaceutical University, Nanjing 211198, PR China

* Corresponding authors.

E-mail addresses: wangxinyu@jsinm.org (Xinyu Wang), gjwang@cpu.edu.cn (Guangji Wang), yangmin@jsinm.org (Min Yang).

*Cell viability of ^89^Zr-labeled and ^68^Ga-labeled cells:*

The ^89^Zr-MSCs and ^68^Ga-MSCs were seeded in 6-well plate (NEST Biotechnology Co.LTD, Wuxi, China) with a density of 1×10^5^ cells per well. The experiment was carried out after cells attaching the wall. The cells were digested into cell suspension using 0.25% trypsin (Beyotime Biotechnology, Shanghai, China), and the 50 μL cell suspension was evenly mixed with Trypan blue staining solution (Beyotime Biotechnology, Shanghai, China), and then a fully automated cell counter (BodBoge, Shenzhen, China) was employed at different time points to detect the viability of the radiolabeled and unlabeled MSCs.

*Cell proliferation of ^89^Zr-labeled and ^68^Ga-labeled cells:*

The ^89^Zr-MSCs and ^68^Ga-MSCs were seeded in 96-well plate with a density of 2000 cells per well. Cells were incubated in an incubator for a set time. The supernatant in the 96 well cell culture plate was discarded, 90 μL serum-free medium and 10 μL Enhanced Cell Counting Kit-8 (Beyotime Biotechnology, Shanghai, China) were added to each well and placed in the incubator for 4 h. OD values were measured with BioTek epoch.

*Cell senescence of ^89^Zr-labeled and ^68^Ga-labeled cells:*

Senescence β-Galactosidase Staining Kit (Beyotime Biotechnology, Shanghai, China) was used to assess cell senescence. Briefly, the culture supernatant in the 6-well plates was removed and the cells were washed once with PBS. The cells were then fixed by incubating with 1 mL of β-galactosidase staining fixative solution for 15 minutes at room temperature. After discarding the fixative, a β-galactosidase staining working solution consisting of X-Gal, and solutions A, B, and C was added. The plates were incubated overnight at 37°C to allow senescent cells to be stained blue by the X-gal chromogenic substrate. Following incubation, the stained cells were visualized under an optical microscope to identify and quantify senescent cells.

*Cell phenotypic function of ^89^Zr-labeled and ^68^Ga-labeled cells:*

In order to study whether the radiolabeled cell has an effect on the phenotypic function of the cell, the gene expression of phenotypic markers was determined by RT-qPCR after radiolabeling. MSCs with three types of unlabeled, ^89^Zr-labeled and ^68^Ga-labeled were collected, and Trizol reagent were added to extract total RNA from the cells according to the kit instructions. Reverse transcription of the extracted RNA using the reverse transcription kit (PrimeScript™ RT reagent Kit (Perfect Real Time), TaKaRa) on the PCR amplification machine (2720 Thermal Cycler, Applied Biosystems, USA). RT-qPCR was then performed to measure mRNA levels of phenotypic markers CD73, CD90, and CD105 using RT-qPCR system (ABI7500, Thermo Fisher, USA) with PCR reaction kit (TB Green® Premix Ex Taq™ II (Tli RNaseH Plus), TaKaRa, Japan). Primer sequences were listed in Table S1.


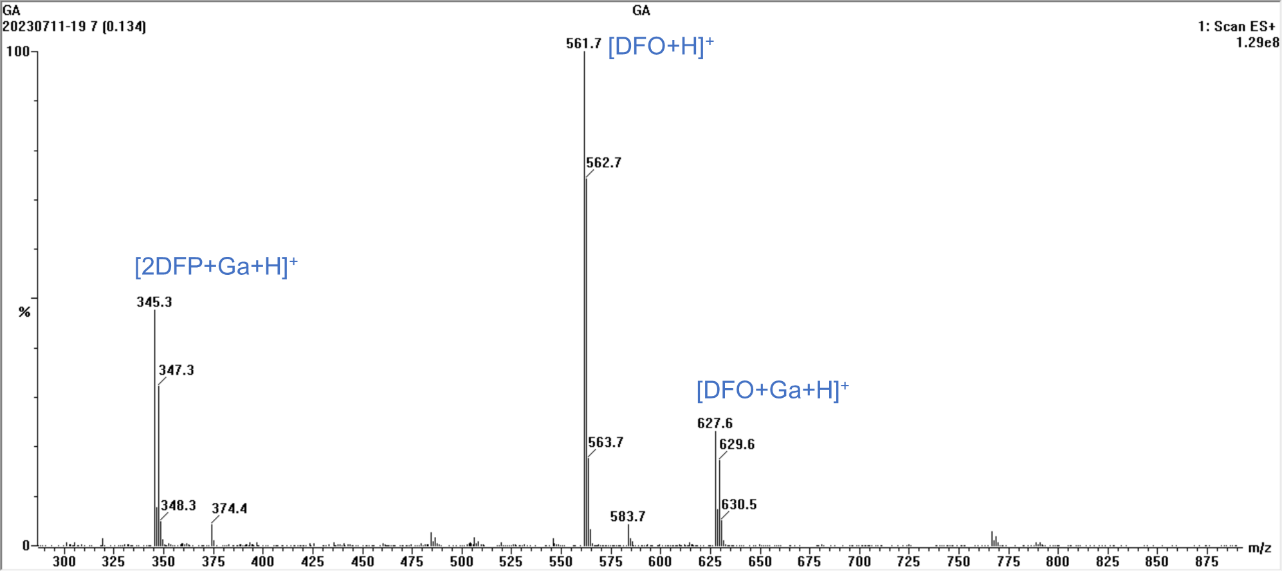


**Figure S1**. ESI mass spectrum of mixture of Ga ions, DFO, DFP, and DFS with a molar ratio of 1:1:1:1.


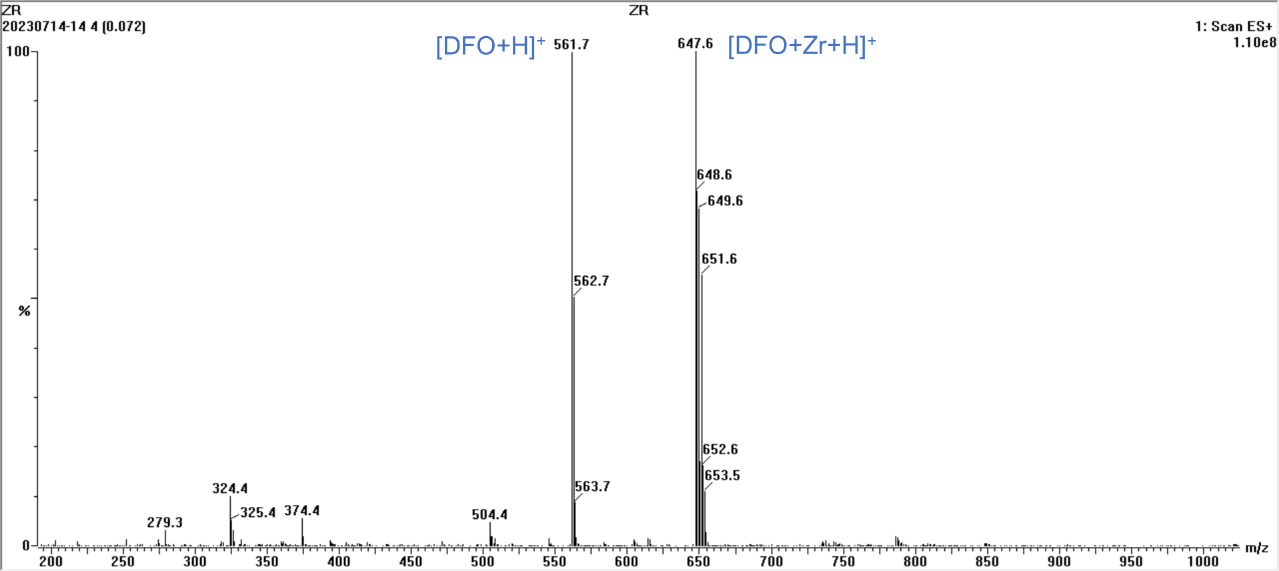


**Figure S2**. ESI mass spectrum of mixture of Zr ions, DFO, DFP, and DFS with a molar ratio of 1:1:1:1.


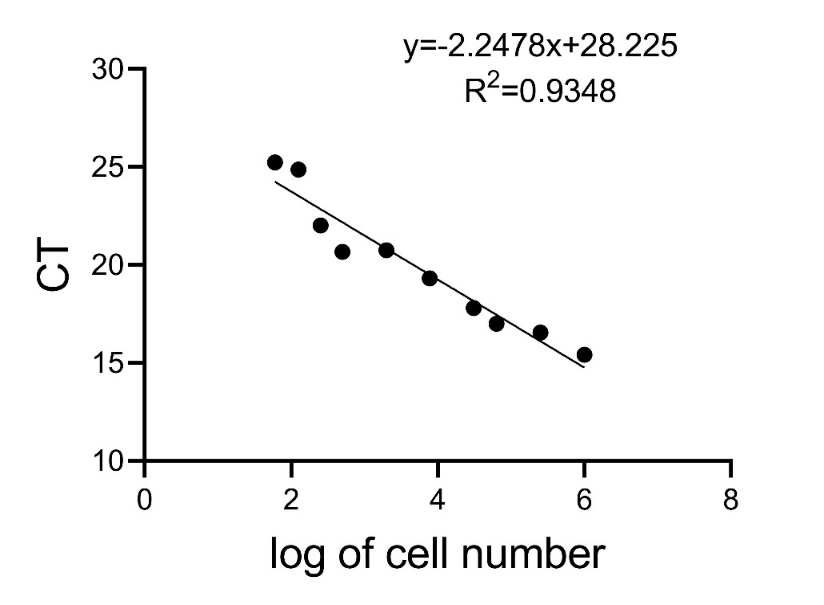


**Figure S3**. Standard curve between cell counts and CT values for qPCR.


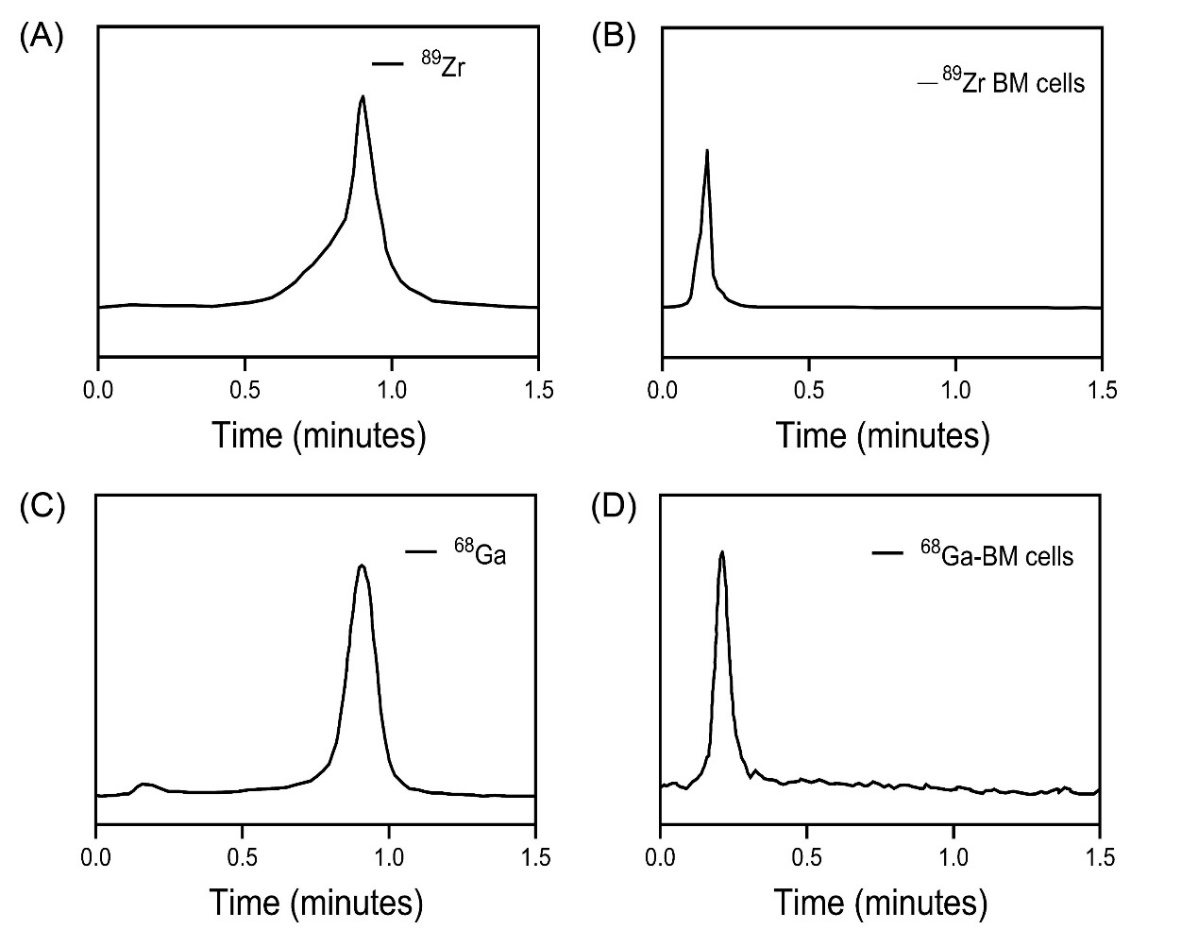


**Figure S4**. The iTLC analysis of free ^89^Zr, ^89^Zr-labeled bone marrow, free ^68^Ga, and ^68^Ga-labeled bone marrow.


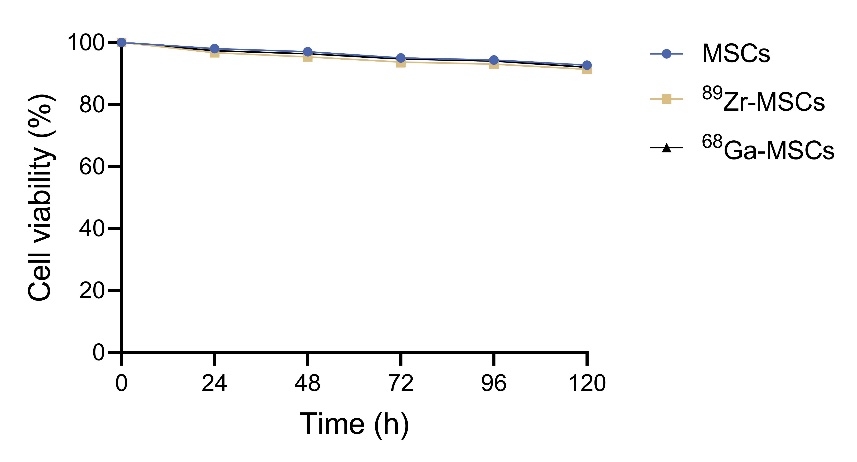


**Figure S5**. Cell viability of ^89^Zr-labled MSCs and ^68^Ga-labled MSCs compared with untreated MSCs determined by trypan blue staining assay. Values are expressed as the means ± SD (n = 3).


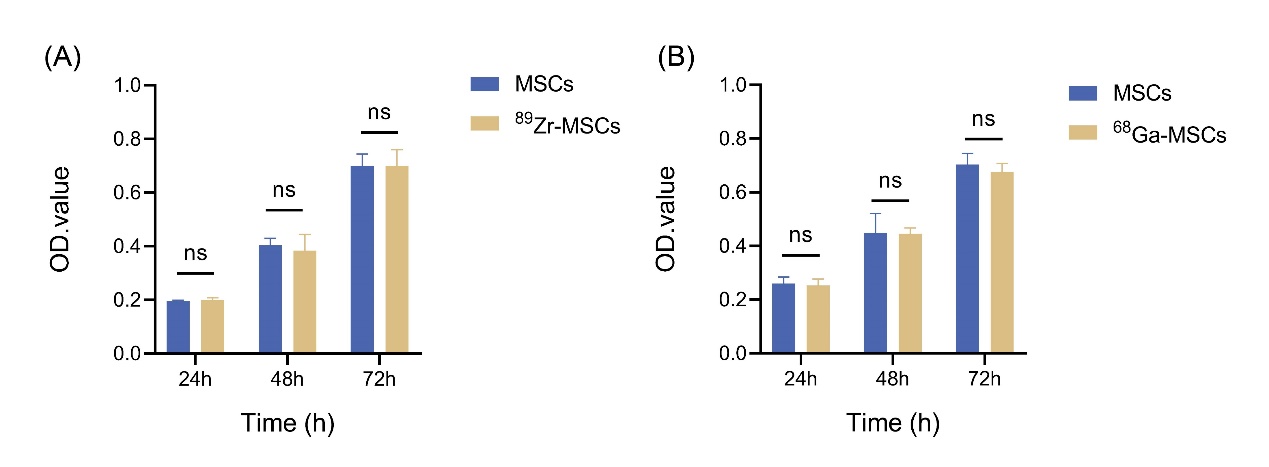


**Figure S6**. Cell proliferation of ^89^Zr-labled MSCs (A) and ^68^Ga-labled MSCs (B) compared with untreated MSCs determined by CCK-8 assay. Values are expressed as the means ± SD (n = 4). ns represents nonsignificant.


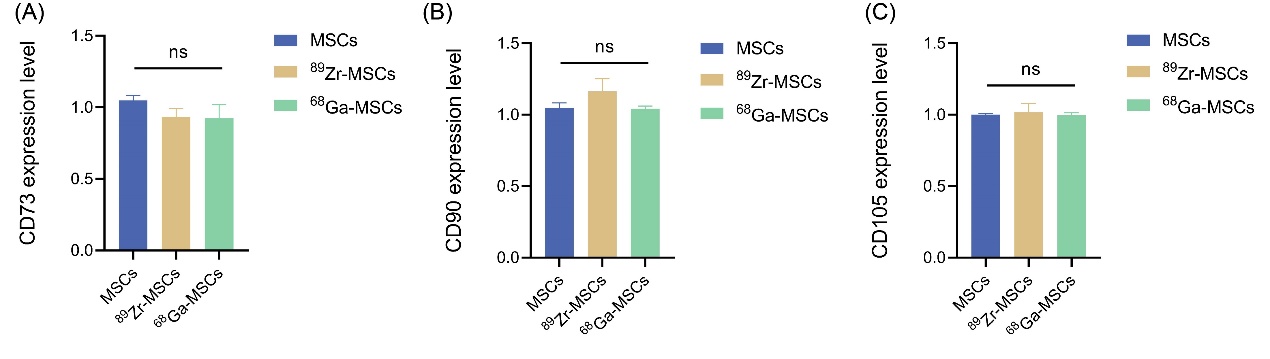


**Figure S7**. Gene expression of MSC marker CD73 (A), CD90 (B), and CD105 (C) in ^89^Zr-labled MSCs and ^68^Ga-labled MSCs determined by RT-qPCR. Values are expressed as the means ± SD (n = 3). ns represents nonsignificant.


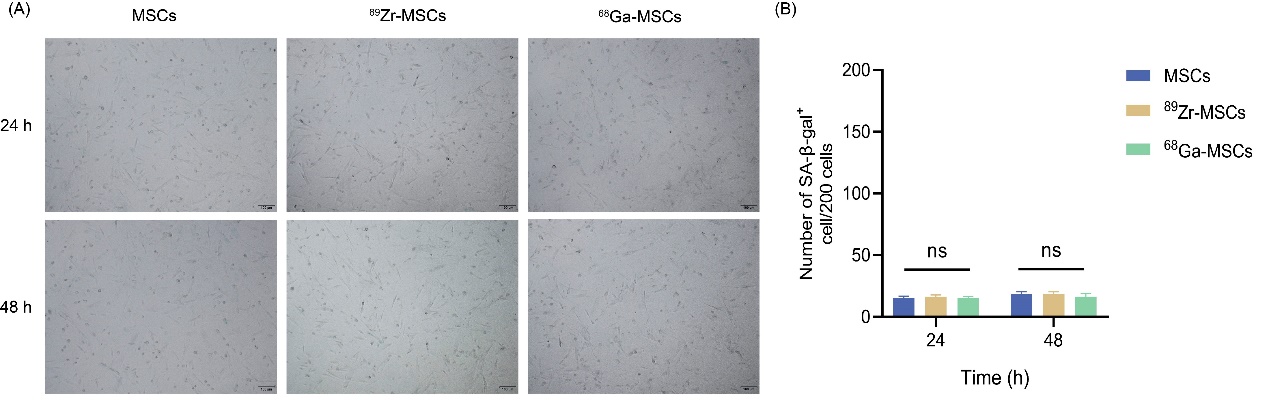


**Figure S8**. (A) β-galactosidase images of ^89^Zr-labled MSCs and ^68^Ga-labled MSCs compared with untreated MSCs. (B) Number of senescence-associated β-galactosidase positive (SA-β-gal^+^) cells (senescent cells) of ^89^Zr-labled MSCs and ^68^Ga-labled MSCs compared with untreated MSCs. Values are expressed as the means ± SD (n = 3). ns represents nonsignificant.

**Table S1**. Primer sequences used for RT-qPCR.

| Genes | Forwards（5`~3`） | Reverse（5`~3`） |
| --- | --- | --- |
| CD73 | ATGTGCCTTTGATGAGTCAGGTAGC | CCCTTCCTTTCTCTCGTGTCCTTTG |
| CD90 | CCAACTTCACCAGCAAATACAA | ACTTGACCAGTTTGTCTCTGAG |
| CD105 | CTTCATGCGCTTGAACATCATC | GAGTAGATGTACCAGAGTGCAG |
| hAlu | CTTGCAGTGAGCCGAGATT | GAGACGGAGTCTCGCTCTGTC |
| β-actin | TCAGCAATGCCTGGGTACAT | ATCACTATTGGCAACGAGCG |

**Table S2**. Ex vivo biodistribution of intravenously transplanted ^89^Zr-labeled MSCs in C57/BL6 mice at different times without or with DFO treatment, respectively.

|  | | | | [^89^Zr]MSCs | | | [^89^Zr]MSCs+DFO | | | | | |
| --- | --- | --- | --- | --- | --- | --- | --- | --- | --- | --- | --- | --- |
|  | 2h | 8h | 24h | | 72h | 108h | | 2h | 8h | 24h | 72h | 108h |
| Blood | 6.3±1.78 | 6.2±0.7 | 2.88±1.29 | | 0.50±0.48 | 0.10±0.08 | | 4.83±1.36 | 0.59±0.20 | 0.13±0.11 | 0.06±0.08 | 0.06±0.05 |
| Heart | 1.03±0.45 | 1.57±0.18 | 1.21±0.40 | | 0.79±0.41 | 0.74±0.66 | | 0.86±0.47 | 0.49±0.11 | 0.75±0.26 | 0.39±0.13 | 0.30±0.10 |
| Liver | 15.52±6.09 | 29.23±1.43 | 37.93±17.87 | | 29.13±5.32 | 21.17±9.68 | | 17.28±3.85 | 26.35±13.41 | 41.55±4.33 | 23.35±7.9 | 11.07±4.02 |
| Spleen | 18.82±15.98 | 42.01±9.26 | 48.99±24.80 | | 37.01±6.30 | 24.44±11.88 | | 15.16±3.57 | 31.73±16.34 | 46.93±9.37 | 26.83±3.97 | 13.74±2.95 |
| Lung | 183.15±111.16 | 64.45±17.42 | 41.72±18.19 | | 23.98±4.25 | 17.04±6.63 | | 172.83±32.68 | 53.10±29.20 | 34.22±11.65 | 22.36±4.13 | 8.87±4.62 |
| Kidney | 7.28±2.51 | 17.27±0.42 | 22.74±12.51 | | 13.49±2.97 | 9.42±3.25 | | 7.78±1.02 | 14.23±5.69 | 17.05±1.18 | 10.01±2.14 | 7.66±2.75 |
| Stomach | 2.12±2.10 | 1.34±0.18 | 1.29±0.60 | | 0.66±0.28 | 0.61±0.33 | | 1.04±0.39 | 0.52±0.20 | 0.60±0.03 | 0.46±0.06 | 0.38±0.20 |
| Intestine | 0.72±0.49 | 1.27±0.65 | 1.19±0.73 | | 0.54±0.28 | 0.58±0.25 | | 0.44±0.13 | 0.48±0.34 | 0.94±0.25 | 0.44±0.17 | 0.29±0.18 |
| Colon | 0.58±0.31 | 0.79±0.18 | 1.25±0.67 | | 0.82±0.57 | 0.56±0.31 | | 0.49±0.19 | 0.60±0.50 | 0.66±0.41 | 0.47±0.37 | 0.22±0.13 |
| Bladder | 0.70±0.32 | 2.11±0.50 | 2.24±1.14 | | 1.31±0.45 | 1.36±1.30 | | 0.80±0.24 | 0.82±0.41 | 1.93±1.20 | 1.19±0.26 | 1.55±1.26 |
| Pancreas | 0.77±0.44 | 1.18±0.47 | 1.56±0.40 | | 0.56±0.10 | 0.53±0.38 | | 0.82±0.23 | 0.46±0.16 | 0.66±0.54 | 0.44±0.18 | 0.67±0.93 |
| Gonad | 0.87±0.40 | 1.48±0.40 | 2.24±0.87 | | 0.77±0.16 | 0.83±0.89 | | 0.83±0.45 | 0.90±0.34 | 0.72±0.24 | 0.69±0.22 | 1.78±2.36 |
| Muscle | 0.31±0.12 | 0.57±0.18 | 1.03±0.81 | | 0.28±0.14 | 0.42±0.39 | | 0.44±0.12 | 0.24±0.07 | 0.27±0.27 | 0.13±0.08 | 0.40±0.53 |
| Knee | 3.01±0.83 | 7.90±1.42 | 30.58±7.12 | | 25.9±10.92 | 19.02±3.92 | | 2.75±0.70 | 4.93±1.57 | 10.56±2.85 | 9.78±2.74 | 4.34±2.11 |
| Femur | 2.22±0.57 | 4.62±0.66 | 16.89±8.36 | | 12.75±3.63 | 13.36±4.78 | | 1.40±0.29 | 2.48±0.84 | 8.17±4.56 | 6.74±2.58 | 2.49±1.88 |
| Tibia | 0.87±0.42 | 3.79±0.66 | 10.89±4.93 | | 10.49±2.59 | 10.67±5.10 | | 1.24±0.16 | 2.30±0.97 | 5.24±1.36 | 4.60±0.97 | 2.33±1.75 |
| Lumbar vertebrae | 2.54±1.17 | 6.01±0.41 | 14.62±6.43 | | 14.18±6.70 | 11.12±3.47 | | 2.32±0.31 | 3.14±1.43 | 6.53±0.82 | 5.66±0.52 | 2.21±1.08 |
| Marrow | 2.46±1.11 | 10.24±4.32 | 15.96±6.08 | | 12.96±2.59 | 9.42±5.24 | | 1.62±0.51 | 11.81±9.17 | 10.57±2.12 | 7.28±1.70 | 3.61±4.20 |
